# Harnessing mRNA technology for ischemic heart disease: a review of regenerative and protective therapies

**DOI:** 10.1097/CP9.0000000000000118

**Published:** 2025-06-30

**Authors:** Matthew Adjmi, Isabelle Tse, Lior Zangi

**Affiliations:** 1 Cardiovascular Research Institute, Icahn School of Medicine at Mount Sinai, New York, New York 10029, USA.; 2 Department of Genetics and Genomic Sciences, Icahn School of Medicine at Mount Sinai, New York, New York 10029, USA.; 3 Black Family Stem Cell Institute, Icahn School of Medicine at Mount Sinai, New York, New York 10029, USA.

**Keywords:** mRNA, Ischemic heart disease, Myocardial infarction, Angiogenesis

## Abstract

As ischemic heart disease (IHD) remains the leading cause of mortality worldwide, there is an urgent need for innovative therapies that go beyond symptom management. The irreversible damage to cardiac tissue following myocardial infarction (MI) and the limited regenerative and proliferative capacity of adult cardiomyocytes (CMs) present significant challenges to the development of treatments capable of restoring cardiac function. This review focuses on emerging modified and non-modified messenger ribonucleic acid (mRNA)-based therapies, which offer targeted and transient protein expression. The studies reviewed here address three major therapeutic strategies: cardiac regeneration, aimed at inducing CM proliferation to restore lost cardiac muscle; cardiac protection, centered on anti-apoptotic and anti-inflammatory methods to mitigate further tissue damage; and cardiovascular regeneration, focused on promoting angiogenesis and restoring vascular integrity after injury. By examining mRNA and modified mRNA (modRNA) therapies across these three approaches, this review showcases mRNA’s promising role in advancing muscular and vascular regenerative and protective therapeutics for IHD.

## INTRODUCTION

Ischemic heart disease (IHD) has been the leading cause of mortality worldwide for over 30 years^[1]^. The burden of IHD continues to grow, driven by the rising prevalence of obesity, diabetes, and hypertension: the major risk factors for the cardiovascular disease (CVD)^[2]^. IHD results from reduced blood flow to the cardiac muscle, often leading to myocardial infarction (MI) due to blockage of the coronary artery. If not treated immediately, MI can lead to heart failure (HF) through ischemia and cell death in the affected area of the heart. The best current treatment for MI is coronary artery reperfusion, wherein blood flow is mechanically restored to cardiac muscle through percutaneous coronary intervention methods, such as balloon angioplasty or stent implantation^[3]^. Although reperfusion significantly improves survival rates, there is a great need for therapies with the ability to regenerate and revascularize the heart in order to restore heart function.

One of the major challenges in treating ischemic injury is the heart’s limited regenerative capacity. The mammalian heart can regenerate for a short window during development and through the first week post-partum. Cardiomyocytes (CMs), the contractile cells of the heart, can proliferate in this window, but shortly after birth, they lose this capacity due to cell-cycle arrest^[4]^. Because the heart cannot regenerate, injury to the heart can be life-threatening.

Ischemic injury leads to multiple, additive effects that deteriorate cardiac function and health. During ischemic injury, large numbers of CMs die, partially reducing overall cardiac contractility. Cardiac remodeling ensues for several weeks after injury, as damaged tissue is replaced with collagen-rich extracellular matrix (ECM), generated by fibroblasts and myofibroblasts, then giving way to non-contractile cardiac scar tissue^[5]^. The deposited ECM restores structural integrity and maintains ventricle thickness^[5–6]^, but reduces cardiac function. Additionally, ceramides, simple membrane sphingolipids, accumulate in the heart during MI^[7–9]^. Elevated intracellular ceramide levels can trigger programmed cell death, and ceramide accumulation onset by MI is associated with a higher probability of MI recurrence and consequent death^[10]^. These structural changes following ischemic injury critically alter heart health and function.

Although reperfusion and standard therapies address acute blockage and its immediate consequences, they do little to repair the damaged heart or restore lost cardiac tissue. Current treatments cannot reverse the loss of functional CMs, nor can they meaningfully promote regeneration or mitigate long-term remodeling. This unmet need has driven growing interest in novel therapeutic strategies that aim to enhance cardiac repair at the cellular level. Among these, messenger ribonucleic acid (mRNA)-based therapies have emerged as particularly promising candidates, offering the ability to transiently express regenerative, protective, or angiogenic proteins directly in cardiac tissue.

This review focuses on emerging modified and non-modified mRNA-based therapies, which offer targeted and transient protein expression.

## STUDY SELECTION

This review includes studies published between 2013 and 2024, identified through PubMed and Scopus searches using keywords such as “mRNA therapy,” “ischemic heart disease,” and “cardiac regeneration.” Studies were included if they proved to focus on mRNA-based approaches (both modified and non-modified) for therapeutic applications in IHD, with outcomes on cardiac regeneration, protection, and/or vascular repair. Non-peer-reviewed publications and those lacking specific therapeutic data were excluded.

## PROTEIN REPLACEMENT THERAPY FOR IHD

In the context of IHD, treatment approaches should aim to induce CM proliferation, prevent cardiac cell death, promote angiogenesis, reprogram fibroblasts, and reduce electrophysiological abnormalities. The most promising non-viral method of delivering genes to the heart is lipid-encapsulated nucleic acid therapeutics, particularly modified mRNA (modRNA) due to its ability to transiently express therapeutic proteins with high efficiency and low immunogenicity.

### mRNA

mRNA is a naturally occurring molecule with great biomedical potential. Endogenous mRNA is transcribed from deoxyribonucleic acid (DNA) and translated into proteins within the cell. Synthetic mRNA can be made in vitro and, when transfected into cells, can be similarly translated into proteins. Accordingly, synthetic mRNA can be used for customizable specific protein replacement therapy that utilizes endogenous cellular functions to allow patients to generate their own personalized treatments. In the first use of synthetic mRNA for protein replacement therapy, as published by Wolff *et al.*^[11]^, synthetic mRNA encoding chloramphenicol acetyltransferase and luciferase were injected and successfully translated in vivo. However, the true potential of mRNA was not yet realized due to its instability and susceptibility to ribonucleases (RNase) and, more importantly, because exogenous mRNA is immunogenic. Delivering mRNA triggers an innate immune response by activating Toll-like receptors (TLRs) which induce inflammation and inhibit protein translation^[12–15]^. For over a decade, these obstacles hampered progress in mRNA protein replacement therapy applications.

### modRNA

In 2005, Dr. Katalin Karikó and Dr. Drew Weissman demonstrated that naturally occurring, chemically modified nucleotides could be used to reduce the immunogenicity of synthetic mRNA^[16–18]^, a breakthrough that later earned them a Nobel Prize. This work was inspired by previous findings that chemically modifying DNA attenuates the immunogenic response to exogenous DNA^[19]^. Applying this approach to in vitro transcribed (IVT) mRNA, Karikó *et al.* queried many nucleoside modifications that could optimize modRNA. Incorporating modified nucleotides such as 5-methylcytidine (m5C), N6-methyladenosine (m6A), 5-methyluridine (m5U), 2-thiouridine (s2U), and pseudouridine (Ψ) decreases RNA recognition by TLRs 3, 7, and 8, thereby reducing the innate immune response in dendritic cells^[16]^. Later experiments showed that Ψ modification exhibited greater translation and biological stability compared to m5C, m6A, m5U, or s2U modifications^[17]^. The advantage of Ψ over the other modified nucleotides relates to reduced activation of RNA-dependent protein kinase (PKR)^[18]^ and resistance to RNaseL^[20]^. Although the field has largely adopted 1-methylpseudouridine (m1Ψ) as the standard modified nucleotide for optimizing mRNA stability and reducing immunogenicity, different combinations of modified nucleotides can have varied effects on translation efficiency, depending on the host cell type^[21]^. modRNA translation efficiency can be augmented by using stable cap analogs^[22]^, G-C enriched constructs, and codon optimization, as well as by optimizing the 5’ and 3’ untranslated regions of the mRNA construct to improve stability and proteins^[23–25]^.

### Methods of delivery

IVT mRNA can be suspended in a sucrose-citrate buffer for storage and unformulated, or “naked,” delivery. Our lab has shown that all types of luciferase mRNA (endogenous mRNA, synthetic mRNA, and modRNA) maintained their integrity for up to 7 days at −20°C or 4°C, or for 1 day at room temperature (RT). These results were confirmed by measuring the bioluminescence of cells transfected with the varying mRNAs under different storage and time conditions^[26]^. Naked delivery presents the simplest way to convey RNA to the heart but requires direct injection to the myocardium. As more clinically feasible alternatives, systemic delivery methods are being studied for cardiac repair. Polymeric delivery techniques utilize natural or synthetic cationic polymers, such as polyethyleneimine (PEI)^[27]^, chitosan^[28]^, plasmid DNA-loaded poly(D,L-lactide-co-glycolide) (PLGA)^[29]^, and dendrimers^[30]^, to protect mRNA and ease its entry into cells. Exosomes, or extracellular vesicles (EVs), have also been evaluated as methods to transport mRNA. EVs are membrane-bound vesicles that shuttle biomolecules and can be synthesized and loaded to encapsulate synthetic modRNA for therapeutic purposes. Nevertheless, the most prevalent method of systemic mRNA delivery remains encapsulation in lipid nanoparticles (LNPs).

### Lipid nanoparticles

mRNA is commonly encapsulated in lipids for intravenous or intra-muscular injection. Due to their inherent hydrophobicity, encapsulation in lipids protects the mRNA from in vivo degradation by ribonucleases and aids in cellular uptake^[31]^. The first successful delivery of liposome-encapsulated mRNA was reported in 1978 in both human^[32]^ and mouse^[33]^ cell lines. Since then, the field has evolved remarkably with optimized lipid encapsulation in treatments for cancer, genetic disorders, and other diseases. Today, lipid encapsulation often employs LNPs that are typically composed of four lipids: an ionizable lipid, cholesterol, a helper lipid, and a polyethylene glycol (PEG). An ionizable lipid is essential for condensing the negatively charged nucleic acid into small particles via charge–charge interactions^[34]^, as well as for interacting with the plasma membrane^[35]^. Cholesterol is used for its rigidity to fill in gaps between lipids, thus contributing to particle stability^[36]^. A neutral helper lipid, such as distearoylphosphatidylcholine (DSPC) or 1,2-dioleoyl-sn-glycero-3-phosphoethanolamine (DOPE), facilitates cellular uptake and endosomal release^[34]^. PEG improves gene transfer, reduces toxicity, and extends circulation time by minimizing the interaction between the particle and the cell surface^[37–38]^.

A major milestone for modRNA-LNP therapeutics is the clinical use in the worldwide vaccination efforts against COVID-19. Both of the predominant mRNA vaccines against COVID-10, Moderna mRNA-1273 and BioNTech/Pfizer BNT162b2, utilize LNP encapsulation. The vaccines initially needed to be stored at −80°C^[39]^, which complicates vaccine transport and accessibility. However, further research and modifications have provided longer shelf life and stability at both 4°C and −80°C. Both of these modRNA-LNP vaccines now use sucrose and Tris-HCL as a cryoprotectant buffer to bolster vaccine stability^[40]^.

### Tissue- and cell-specific delivery

There have been several recent advances that manipulate lipid optimization of organ- and tissue-specific LNPs^[41]^ in order to facilitate either active or passive targeting. Passive targeting utilizes a carefully selected lipid formulation, with differing lipid ratios that will cause particles to accumulate in a specific organ or tissue. Active targeting refers to conjugating small molecules, such as ligands, carbohydrates, peptides, antibodies, or aptamers, onto the particle surface in order to promote its delivery^[42]^. Recent advances in passive LNP targeting of the heart include lipid ratio optimization by Scalzo *et al.*^[43]^ for improved plasmid DNA (pDNA) delivery to CMs, as well as the discovery by Radmand *et al.*^[44]^ that combining cationic cholesterol with a cationic helper lipid enhances heart specificity. However, active targeting approaches specifically designed for cardiac applications remain relatively underdeveloped compared to those for other organs. A notable exception is the study by Rurik *et al.*^[45]^ demonstrating the innovative use of T-cell-targeting LNPs carrying chimeric antigen receptor (CAR) mRNA, which binds to activated fibroblasts, ultimately reducing cardiac fibrosis in a heart disease model.

In the context of IHD, an additional factor may favor LNP accumulation in injured tissue. One study suggests that injury-induced vascular permeability results in leakiness and consequent bioaccumulation of LNPs in damaged myocardium but not in healthy myocardium^[46]^, thereby indicating increased LNP efficiency in an ischemic setting. Although these organs targeting formulation strategies represent important steps toward more selective delivery, further refinement is needed to fully harness their potential in cardiac therapies. Both improved passive formulations and more sophisticated active targeting strategies–integrating multi-ligand surfaces or disease-specific biomarkers–could significantly enhance tissue specificity and therapeutic efficacy from the formulation alone.

**Figure 1.**
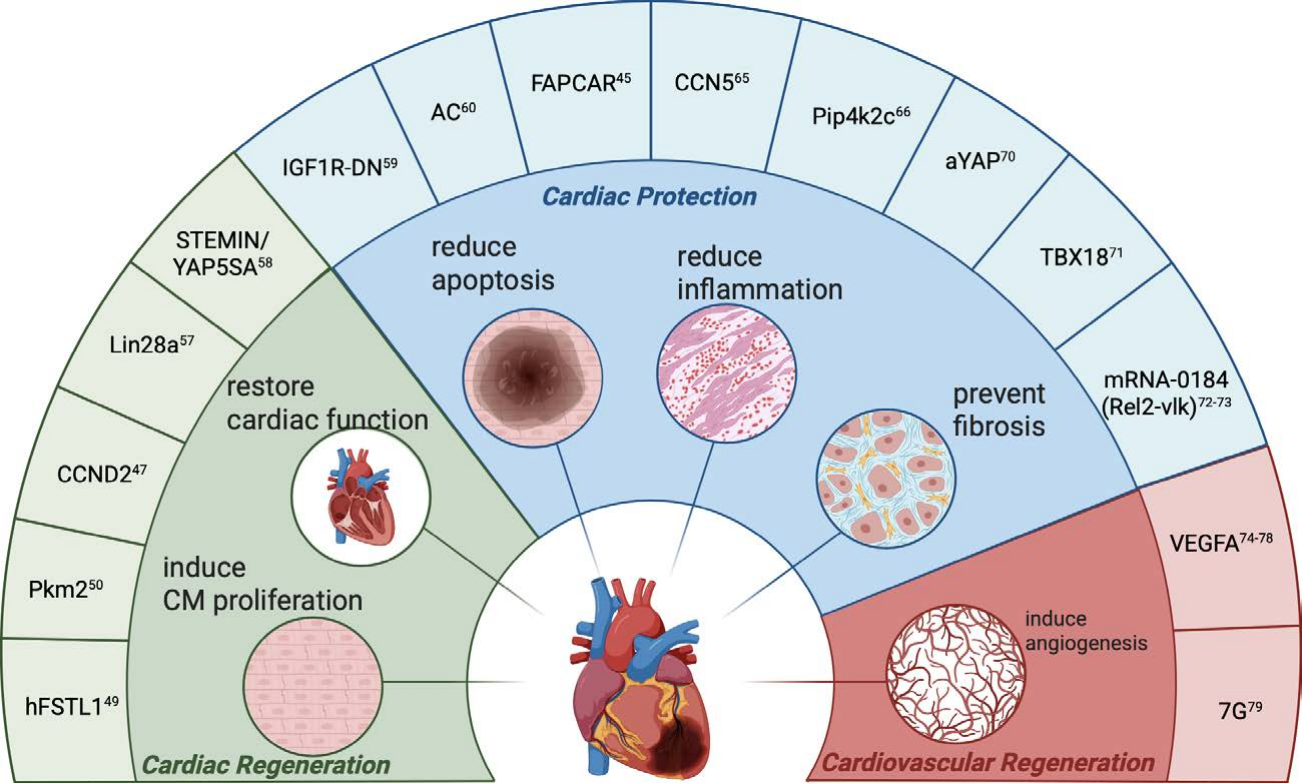
**Graphic summary of genes considered for mRNA therapy in regeneration, protection, and/or revascularization of the heart.** AC: acid ceramidase; aYAP: a constitutively active form of yes-associated protein (YAP); CCN5: cellular communication network factor 5; CM: cardiomyocyte; FAPCAR: the targeted use of fibroblast-activated protein and chimeric antigen receptor mRNA; hFSTL1: human follistatin-like 1; IGF1R-DN: a dominant negative IGF-1 receptor modRNA; Lin28a: lin-28 homolog A; mRNA: messenger ribonucleic acid; Pip4k2c: type 2 phosphatidylinositol-5-phosphate 4-kinase gamma; Pkm2: pyruvate kinase muscle isoenzyme 2; Rel2-vlk: human relaxin-2 fused to a variable light chain kappa; STEMIN1: stem cell-inducing factor 1; TBX18: T-box transcription factor 18; VEGFA: vascular endothelial growth factor A; YAP5SA: an active mutant of the yes-associated protein (YAP).

To enhance the organ and cell-type specificity of modRNA protein replacement therapy, our lab has developed the specific modRNA translation system (SMRTs). This system consists of two modRNA constructs, utilizing L7Ae or Cas-6 as a suppressor gene to limit gene-of-interest expression to a targeted cell type. The first construct codes for the therapeutic protein of interest and contains a suppressor gene binding site, the k-motif for L7Ae or hairpin for Cas-6. The other construct codes for the corresponding suppressor protein by carrying cell-specific microRNA (miR) binding sites^[47]^. Upon SMRTs delivery to the targeted cell type, the endogenous miRs bind and degrade the suppressor construct, thus allowing uninhibited translation of the gene of interest. Upon SMRTs delivery to all other cell types, the suppressor construct will translate, bind, and suppress the gene-of-interest construct. This approach enables precise control over gene expression, thereby permitting therapeutic genes to be active only in targeted cells and minimizing off-target effects. Cell- and organ-specific targeting enhance the safety and efficacy of modRNA therapies (Figure 1).

## CARDIAC REGENERATION

Cardiac regeneration treatments aim to promote CM proliferation and stimulate other repair mechanisms in order to restore function of the heart after ischemic injury. One approach by Magadum *et al.* uses human follistatin-like1 (hFSTL1) modRNA (Table 1, row 1). Though epicardial hFSTL1 supports CM proliferation and cardiac regeneration, myocardial hFSTL1^[48]^ does not. Their discovery describes that a single asparagine-to-glutamine mutation at the N180 glycosylation site of hFSTL1 is necessary for its ability to promote CM proliferation and cardiac regeneration^[49]^. Directly injecting naked, nonglycosylated hFSTL1 modRNA to adult mice seven days post-MI resulted in significantly better cardiac function, expanded capillary density in the left ventricle, and decreased scar size 28 days post-MI^[49]^.

**Table 1.** Preclinical and clinical studies involving mRNA treatment for cardiac regeneration, protection, or revascularization

Gene	mRNA platform	Focus	Animal	Model	Delivery method	Administration	Phase of study	Result	Reference
Nonglycosylated hFSTL1	modRNA: m1Ψ, ARCA, poly-A tail	Cardiac regeneration	Mouse	MI	Naked: sucrose-citrate buffer	Intracardiac	Preclinical	Increased cardiac function and capillary density of the left ventricle, plus decreased scar size	Magadum *et al*.^[49]^
Pkm2	modRNA: m1Ψ, ARCA, poly-A tail	Cardiac regeneration	Mouse	MI	Naked: sucrose-citrate buffer, CM-SMRTs	Intracardiac	Preclinical	Activated ki67 and pH3, and increased capillary density and left ventricle thickness	Magadum *et al*.^[50]^
Cyclin D2 (CCND2)	modRNA: N1-methylpseudouridine	Cardiac regeneration	Mouse and pig	MI	CM-SMRTs	Intracardiac	Preclinical	Upregulated Ki67 and pH3, stimulated CM proliferation, decreased infarct size, and improved cardiac function	Sun *et al*.^[47]^
Lin28a	N/A	Cardiac regeneration	Mouse	MI	CM-SMRTs	Intracardiac	Preclinical	Induced CM division and reduced scar formation through downregulation of Let-7	Magadum *et al*.^[57]^
STEMIN/YAP5SA	modRNA	Cardiac regeneration	Mouse	MI	Naked	Intracardiac	Preclinical	Improved cardiac function and myocardial fibrosis in left ventricles of infarcted adult mice	Xiao *et al*.^[58]^
IGF1R-DN	modRNA: m5C, ΨU, ARCA, poly-A tail, 5’ and 3’ UTRs	Cardiac protection	Mouse	MI	Polyethylenimine-based particles	Intracardiac	Preclinical	Decreased caspase-9 activity and protection from apoptosis	Zangi *et al*.^[59]^
AC	modRNA: m1Ψ, ARCA, poly-A tail	Cardiac protection	Mouse	MI	Naked: sucrose-citrate buffer	Intracardiac	Preclinical	Upregulation of AC and generation of S1P led to increased cell survival in hypoxic and MI models due to lower ceramide levels and cell death rates	Hadas *et al*.^[60]^
FAPCAR	modRNA: m1Ψ, ARCA, poly-A tail, 5’ and 3’ UTRs	Cardiac protection	Mouse	Hypertensive model of cardiac fibrosis and injury	CD5 LNP with modRNA	Intravenous	Preclinical	Reprogramming of T cells to CAR T cells led to reduced fibrosis and improved cardiac function	Rurik *et al*.^[45]^
CCN5	modRNA: m1Ψ, ARCA, poly-A tail, 5’ and 3’ UTRs	Cardiac protection	Mouse	MI	Naked	Intracardiac	Preclinical	Decreased cardiac fibrosis and improved cardiac function by inhibiting myofibroblast transdifferentiation and inducing apoptosis of fibroblasts	Song *et al*.^[65]^
Pip4k2c	modRNA: m1Ψ, ARCA, poly-A tail	Cardiac protection	Mouse	TAC model	Naked: sucrose-citrate buffer	Intracardiac	Preclinical	Upregulation of Pip4k2c increased heart function and survival through reversal of cardiac fibrosis and hypertrophy	Magadum *et al*.^[66]^
aYAP	N/A	Cardiac protection	Mouse	MI	Saline	Intracardiac	Preclinical	Sustained YAP activation led to reductions in CM death	Chen *et al*.^[70]^
TBX18	modRNA: N1-methylpseudouridine-5′-triphosphate	Cardiac protection	Rats and pigs	CAVB	Naked	Intramyocardial	Preclinical	Reprogrammed CMs into pacemaker cells in rats and pigs, improving ventricular function	Wolfson *et al*.^[71]^
mRNA-0184 (Rel2-vlk)	N/A	Cardiac protection	Humans	Chronic heart failure	LNP-modRNA	N/A	Phase I clinical	Ongoing trial	ModernaTX Inc.^[73]^
mRNA-0184 (Rel2-vlk)	N/A	Cardiac protection	Nonhuman primates	Naturally developed cardiovascular and metabolic disease	LNP-modRNA	Intravenous	Preclinical	0.025 mg/kg administered at 2-week intervals achieved optimal relaxin-plasma concentrations (1–2.5 ng/mL)	Kaushal *et al*.^[72]^
VEGFA	m5C, ΨU, ARCA, poly-A tail	Cardiovascular regeneration	Mouse	N/A	Matrigel, modRNA, RNAiMAX, and heart progenitors	Subcutaneous	Preclinical	VEGFA modRNA differentiates Isl1 + progenitors to endothelial cells, and promotes formation of stable vascular structures	Lui *et al*.^[74]^
VEGFA	m5C, ΨU, ARCA, poly-A tail, 5’ and 3’ UTRs	Cardiovascular regeneration	Mouse	MI	RNAiMAX	Intracardiac	Preclinical	VEGFA modRNA redirected progenitor cells to cardiovascular lineages, improving cardiac function and survival post-MI	Zangi *et al*.^[75]^
VEGFA	m5C, ΨU, m1Ψ, poly-A tail, 5’ and 3’ UTRs, methyl group SAM	Cardiovascular regeneration	Pig	MI	Naked: sucrose-citrate buffer	Intracardiac	Preclinical	Single injection of VEGFA modRNA led to increased ejection fraction, enhanced vessel density, and reduced myocardial fibrosis	Carlsson *et al*.^[76]^
sAZD8601 (VEGFA)	N/A	Cardiovascular regeneration	Human	Coronary bypass surgery	Naked: citrate-buffered saline	Epicardial	Clinical	30 epicardial injections of AZD8601 improved ejection fraction and decreased cardiac stress levels without infections, immune responses, or arrhythmias	Anttila *et al*.^[77]^
VEGFA	m1Ψ, poly-A tail, 5’ and 3’ UTRs	Cardiovascular regeneration	Rat	MI	MessengerMAX lipofectamine	Intracardiac	Preclinical	VEGFA production upregulated pro-survival pathways, improving engraftment and left ventricular function post-MI	Ai *et al*.^[78]^
7G-modRNA (Gata4, Mef2c. Tbx5, Hand2, DN-TGFb, DN-Wnt8a, and acid ceramidase)	m1Ψ, ARCA	Cardiovascular regeneration	Mouse	MI	Sucrose-citrate buffer	Intracardiac	Preclinical	Reprogrammed ~25% non-CMs into CMs, upregulated pro-angiogenic pathways, and improved cardiac function, scar size, long-term survival, and capillary density	Kaur *et al*.^[79]^

Ψ: pseudouridine; AC: acid ceramidase; aYAP: a constitutively active form of yes-associated protein (YAP); ARCA: anti-reverse cap analog; CAVB: complete atrioventricular block; CCN5: cellular communication network factor 5; CM: cardiomyocyte; FAPCAR: the targeted use of fibroblast-activated protein and chimeric antigen receptor mRNA; hFSTL1: human follistatin-like 1; IGF1R-DN: a dominant negative IGF-1 receptor modRNA; Lin28a: lin-28 homolog A; LNP: lipid nanoparticle; m5C: 5-methylcytidine; m5U: 5-methyluridine m6A: N6-methyladenosine; modRNA: modified mRNA; MI: myocardial infarction; Pip4k2c: type 2 phosphatidylinositol-5-phosphate 4-kinase gamma; Pkm2: pyruvate kinase muscle isoenzyme 2; Rel2-vlk: human relaxin-2 fused to a variable light chain kappa; RNA: ribonucleic acid; SMRTs: specific modRNA translation system; STEMIN1: stem cell-inducing factor 1; TBX18: T-box transcription factor 18; UTR: untranslated region; VEGFA: vascular endothelial growth factor A; YAP: yes-associated protein; YAP5SA: an active mutant of the yes-associated protein (YAP).

Similarly, the same group demonstrated that pyruvate kinase muscle isoenzyme 2 (Pkm2) modRNA has promising effects in cardiac regeneration^[50]^ (Table 1, row 2). Pkm2 functions by reducing pyruvate kinase activity and promoting the pentose phosphate pathway (PPP), which mitigates oxidative stress^[51–54]^. To enhance CM-specific expression, Pkm2 modRNA was incorporated into CM-specific SMRTs^[50]^ which was developed with miRs shown to be exclusive to CMs, miR1-1^[55]^, and miR208a^[56]^. The Pkm2-CM-SMRTs activated cell-cycle markers Ki67 and pH3, thereby suggesting enhanced CM proliferation. Directly injecting naked Pkm2-CM-SMRTs in acute and chronic MI models increased both capillary density and left ventricle thickness^[50]^. This group also tested CM-SMRTs with other gene targets, such as cyclin D2 (CCND2) and lin-28 homolog A (Lin28a) modRNA, intended to induce cardiac regeneration. Intramyocardial injections of CCND2-CM-SMRTs in mouse and pig MI models upregulated Ki67 and pH3, stimulated CM proliferation, decreased infarct size, and improved cardiac function without raising the risk of arrhythmia^[47]^ (Table 1, row 3). Additionally, Lin28a-CM-SMRTs induced CM division and diminished scar formation in murine MI models^[57]^. The results indicate that Lin28a-mediated downregulation of Let-7 represses cMYC, high mobility group AT-hook 2 (HMGA2), and K-RAS (Table 1, row 4). These experiments showcase the potential of these targets and the specificity of CM-SMRTs for selective CM regeneration and overall cardiac repair with minimal stray effects.

A novel combination approach by Xiao *et al.*^[58]^ explores the use of modRNA-driven cellular reprogramming for cardiac regeneration. In a murine MI model, intramyocardial injection of stem cell-inducing factor 1 (STEMIN) and YAP5SA, an active mutant of the yes-associated protein, modRNA promoted the upregulation of cell-cycle genes and nuclear replication in CMs (Table 1, row 5). STEMIN, a serum response factor (SRF) mutant, reprograms CMs to exhibit more stem-like properties, while YAP5SA—a Hippo pathway inhibitor—drives CM proliferation. This therapy resulted in significantly improved left ventricular function, reduced fibrosis, and increased myocardial wall thickness. As with all mRNA therapeutics, the transient nature of modRNA expression enables controlled cardiac reprogramming without long-term genomic risks, making it a promising strategy for advancing regenerative treatments in IHD.

### Overview

hFSTL1 modRNA enhances cardiac regeneration specifically when delivered to the epicardium, with clear evidence of improved function, neovascularization, and scar reduction after MI. Pkm2, CCND2, and Lin28a modRNAs—delivered via CM-specific SMRTs—demonstrate robust regenerative effects across both small and large animal models, showing increased CM proliferation, reduced infarct size, and preserved heart function with minimal off-target effects. The STEMIN/YAP5SA modRNA combination directly stimulates CM reprogramming and proliferation in vivo, demonstrating strong regenerative potential while avoiding permanent genomic modification due to the transient nature of modRNA expression. Together, these studies demonstrate that mRNA therapies targeting CM proliferation—whether through enhancing endogenous regenerative signaling, optimizing metabolic state, or directly reprogramming cell fate—have shown clear evidence of improved cardiac function, reduced fibrosis, and increased vascularization across multiple preclinical models. These findings underscore the versatility of mRNA-based approaches for cardiac regeneration and highlight their potential as a foundation for future translational strategies in IHD.

## CARDIAC PROTECTION

Cardiac protection therapies are intended to protect cardiac tissue from injury or stress by attenuating inflammation, fibrosis, oxidative stress, or apoptosis. Several approaches have shown promise in achieving these goals through targeted intervention. One such method employs insulin-like growth factor 1 (IGF-1) modRNA, which has been shown to effectively reduce CM apoptosis when injected intracardially immediately after left anterior descending artery (LAD) ligation in a murine model (Table 1, row 6). IGF-1’s anti-apoptotic capacity hinges on its ability to expand phosphorylation of Akt and curtail caspase-9 activity^[27]^. However, our group has shown that delivering IGF-1 modRNA to the heart post-MI causes epicardial-derived cells to differentiate into fat cells^[59]^, an outcome that leads to detrimental epicardial adipose tissue (EAT) formation. To counteract this effect, we developed a dominant negative IGF-1 receptor modRNA (IGF1R-DN) to inhibit the IGF-1 signaling pathway, thereby preventing EAT formation and enhancing cardiac protection post-MI^[59]^.

Another cardiac protection effort involves administering acid ceramidase (AC) modRNA to modulate lipid metabolism. In both humans and rodents, the post-MI increase in cardiac ceramide levels is associated with apoptosis in the left ventricle and decreased cardiac function^[7–9]^. Hadas *et al.* used AC-modRNA to reduce ceramide buildup in cardiac tissue post-MI^[60]^. AC catalyzes ceramide hydrolysis to free fatty acids and sphingosine, which is then phosphorylated by Sphk to generate S1P, a pro-survival molecule^[61–63]^. This work elucidates two potential targets, AC and Sphk1, that may protect the heart: treatment with AC, Sphk1, or AC + Sphk1 modRNA significantly improved heart function and reduced scar size in an MI mouse model (Table 1, row 7).

As previously mentioned, the study by Rurik *et al.*^[45]^ utilized fibroblast activation protein (FAP) CAR modRNA to engineer transient CAR T cells to selectively target and eliminate activated fibroblasts, thereby reducing fibrosis in the context of cardiac injury (Table 1, row 8). Deploying modRNA in a transient CAR T-cell approach sidesteps the risks associated with persistent CAR T-cell presence, such as over-destruction of cardiac fibroblasts. The group used CAR modRNA designed against FAP encapsulated in an antibody-conjugated LNP to actively target CD5 cells. The resulting CAR T cells effectively limited fibrosis and promoted cardiac function in a murine hypertensive model of cardiac fibrosis and injury^[45]^.

Another target that could attenuate injury-associated fibrosis in the heart is cellular communication network factor 5 (CCN5), also known as WNT1-inducible signaling pathway protein 2 (WISP-2), which can impede the pro-fibrotic connective tissue growth factor (CTGF/CCN2)^[64]^. Song *et al.*^[65]^ showed the antifibrotic properties of CCN5 modRNA in both preventative and therapeutic murine MI models, in which CCN5 modRNA was injected intracardially either immediately or 2 weeks after MI, respectively (Table 1, row 9). Their findings revealed that CCN5 modRNA effectively blocked myofibroblast transdifferentiation and induced the apoptosis of existing fibroblasts, thus lowering and even preventing cardiac fibrosis in both models without causing LV rupture^[65]^.

Magadum *et al.*^[66]^ have explored the cardioprotective capability of administering type 2 phosphatidylinositol-5-phosphate 4-kinase gamma (Pip4k2c) to inhibit transforming growth factor beta 1 (TGFB1) and the mechanistic target of rapamycin complex 1 (MTORC1) (Table 1, row 10). In a mouse model of pressure overload by transverse aortic constriction (TAC), directly injecting Pip4k2c modRNA was shown to restrain fibrosis and boost ejection fraction and fractional shortening, as compared to outcomes in untreated control TAC heart^[66]^. Similarly, modulating Yes-associated protein (YAP) has been studied as a cardioprotective therapy. Through its capacity to suppress TLRs, YAP has been shown to regulate immune responses and cell survival and thus may be able to reduce cardiac inflammation and improve cardiac outcomes after MI^[67–69]^. Chen *et al.*^[70]^ have shown that modRNA of a constitutively active form of YAP (aYAP), delivered intracardially, can significantly reduce CM death in a mouse model of myocardial ischemia-reperfusion (Table 1, row 11). Furthermore, they emphasized that transient gene expression achieved through modRNA can advantageously avoid the oncogenic risks associated with sustained YAP activation.

Building on efforts to develop biological alternatives to electronic pacemakers, Wolfson *et al.*^[71]^ report the use of T-box transcription factor 18 (TBX18) mRNA to reprogram CMs to pacemaker cells (Table 1, row 12). Embryonic TBX18 is required for the development of pacemaker cells during embryonic development. This study reports the ability of transient TBX18 expression, via direct myocardial injection of naked TBX18 mRNA, to create a biological pacemaker in complete atrioventricular block (CAVB) rat and pig models. Pigs treated with TBX18 mRNA exhibited heartbeats that responded to natural variations in activity, while control pigs remained dependent on an implanted pacemaker. Over 4 weeks, treated pigs exhibited increased heart rate variability, better ventricular synchrony, and improved autonomic heart rate control. These results show that a single dose of TBX18 mRNA can generate sustained ventricular pacing and maintain physiologic heart rate responsiveness, presenting a promising alternative to electronic pacemakers used to treat bradycardia, atrioventricular block, and HF^[71]^.

Currently in a phase I clinical trial (Table 1, row 13), Kaushal *et al.*^[72]^ reports a preclinical pharmacokinetic/pharmacodynamic (PK/PD) study in nonhuman primates of Moderna’s mRNA-0184 to extrapolate a safe starting dose. mRNA-0184 is an LNP-encapsulated mRNA therapy encoding human relaxin-2 fused to a variable light chain kappa (Rel2-vlk). Relaxin-2 is a naturally occurring peptide hormone with vasodilatory, antifibrotic, and anti-inflammatory properties, making it a promising cardioprotective agent. Relaxin-based therapies have been heavily studied and developed by many groups to treat HF, such as current trials with AZD3427 and AZD5462 by AstraZeneca, but mRNA-0184 is the only mRNA-based relaxin therapy in clinical trials.

The (PK/PD) study of mRNA-0184 demonstrated dose-dependent expression of the Rel2-vlk protein with therapeutic levels maintained for several days post-intravenous delivery into cynomolgus monkeys with naturally developed CVD (Table 1, row 14). The study identified a starting dose of 0.025 mg/kg administered at 2-week intervals for the phase I trial in humans (NCT05659264^[73]^). This dose was determined by the efficiency to achieve relaxin-plasma concentration (1–2.5 ng/mL) associated with enhanced cardiac output, reduced systemic vascular resistance, and attenuation of myocardial hypertrophy and fibrosis^[72]^.

### Overview

Pathway modulation using the IGF1R-DN modRNA strategy was able to enhance cardiac protection post-MI, while preventing EAT formation associated with IGF-1. Targeting ceramide metabolism through AC and Sphk1 modRNA offers a novel metabolic approach to cardiac protection, highlighting the importance of addressing ceramide-driven apoptosis post-MI. The FAPCAR approach demonstrates how modRNA can enable transient, cell-targeted immunotherapy to mitigate fibrosis while avoiding the chronic risks of persistent CAR T-cell activity. CCN5 modRNA directly targets fibrotic remodeling by modulating fibroblast activity, providing a promising tool for both early and delayed antifibrotic therapy post-MI. Pip4k2c and aYAP modRNA therapies show the potential to mitigate both fibrotic remodeling and inflammation-driven CM death, underscoring the importance of targeting pro-fibrotic and immune signaling cascades in cardiac protection. TBX18 mRNA successfully reprogrammed CMs into pacemaker cells, demonstrating the feasibility of creating a biological pacemaker with adaptive heart rate responsiveness—a critical advance for treating bradycardia and heart block. mRNA-0184 demonstrates how mRNA therapeutics can deliver long-acting, cardioprotective hormones. Together, these cardiac protection therapies illustrate the versatility of mRNA therapeutics in addressing multiple mechanisms of cardiac injury, from apoptosis and fibrosis to metabolic dysfunction and electrophysiological disorders. The ability to target diverse pathways with transient, tunable expression highlights mRNA’s adaptability for both acute and chronic cardiac protection in IHD and HF.

## CARDIOVASCULAR REGENERATION

Cardiovascular regeneration strategies encompass therapeutic options designed to restore vascular integrity, promote angiogenesis, and enhance blood flow to ischemic tissues. One promising target in these efforts is vascular endothelial growth factor A (VEGFA), which has been studied extensively in this context. Lui *et al*.^[74]^ demonstrated that VEGFA modRNA was able to both direct human Isl1 + progenitors toward an endothelial fate as well as significantly strengthen their engraftment, proliferation, and survival (Table 1, row 15). When delivered subcutaneously in a modRNA-transfected Matrigel mixture, VEGFA modRNA differentiated Isl1 + progenitors into cardiac endothelial cells, thereby supporting the formation of stable vascular structures in NOD/SCID mice.

Building on previous work on VEGFA, our group, Zangi *et al.*^[75]^ further explored the regenerative effects of VEGFA modRNA in a murine MI model. Indeed, we were able to show that VEGFA modRNA can mobilize epicardial progenitor cells and shift them toward cardiovascular lineages. VEGFA modRNA thus produced stable vessels with normal permeability and, consequently, boosted long-term cardiac function and significantly improved survival 1-year post-MI (Table 1, row 16)^[75]^. Carlsson *et al.*^[76]^ applied our results to large animals by applying injected VEGFA 165 modRNA to a porcine MI model. A single intracardial injection administered 1-week post-infarction increased ejection fraction, expanded vessel density in the injury border zone, and decreased myocardial fibrosis over a 2-month period (Table 1, row 17). The group also determined that VEGFA modRNA did not activate innate immunity in monkeys or rats when injected intradermally or intravenously^[76]^.

The EPICCURE phase 2a trial, AZD8601, evaluated a modified mRNA therapeutic encoding VEGFA for safety and efficacy as a treatment for patients with IHD undergoing coronary artery bypass grafting (Table 1, row 18). The group opted to inject the drug naked, suspended in a citrate-saline solution. Patients underwent 30 epicardial injections into pre-mapped ischemic myocardial regions, administered immediately after bypass grafting. Each patient in the AZD8601 group received a total dose of 3 mg modRNA. Compared to placebo, the treatment group exhibited a trend toward improved ejection fraction as well as lower levels of N-terminal pro-B-type natriuretic peptide (NT-proBNP) levels, a cardiac biomarker indicative of stress^[77]^.

Several other groups have explored a variety of VEGFA modRNA-based treatment methods. Ai *et al.*^[78]^ utilized a cell therapy approach to cardiovascular regeneration by preconditioning iPSC-derived CMs (iPSC-CMs) with VEGFA modRNA before transplanting them into a rat MI model (Table 1, row 19). The VEGF-treated cells upregulated pro-survival pathways, thereby augmenting engraftment and left ventricular function, enhancing cell survival and neovascularization, and limiting remodeling and fibrosis. These results underscore the potential of VEGF modRNA in cardiac repair^[78]^. Kaur *et al.*^[79]^ employed a reprogramming technique with a 7-gene modRNA cocktail (7G), combining cardiac reprogramming genes (Gata4, Mef2c, Tbx5, Hand2) and reprogramming-helper genes (DN-TGFb, DN-Wnt8a, AC), to induce cardiovascular regeneration (Table 1, row 20). In mice, directly injecting 7G into the scar area reprogrammed approximately 25% of non-CMs (non-CMs) into CMs. The group noted significantly better cardiac function, scar size, long-term survival, and capillary density in treated mice. Although 7G did not produce functional beating CMs, it did promote pro-angiogenic mesenchymal cell markers and the sustained secretion of angiogenic factors, both of which support vascular regeneration in both cardiac and skeletal muscle ischemia models. In vitro studies on human ventricular fibroblasts confirmed 7G-modRNA’s efficacy in upregulating similar pro-angiogenic pathways^[79]^.

### Overview

VEGFA modRNA not only promotes endothelial differentiation but also enhances the engraftment and survival of progenitor cells, demonstrating its potential to foster stable neovascularization. VEGFA modRNA’s ability to mobilize endogenous progenitor cells, promote stable vasculature, and improve long-term cardiac function positions it as a leading candidate for both small and large animal models of cardiovascular regeneration. The EPICCURE trial highlights the clinical feasibility of VEGFA modRNA delivery during surgery, with early signals suggesting potential functional benefit and biomarker improvement in patients with IHD. Preconditioning transplanted cells with VEGFA modRNA enhances their survival and regenerative potential, demonstrating a promising hybrid approach combining gene therapy and cell therapy for IHD. The 7G-modRNA cocktail highlights the potential of direct *in situ* reprogramming to stimulate vascular and myocardial regeneration, even if complete CM maturation is not achieved. Across these diverse approaches, cardiovascular regeneration therapies using modRNA demonstrate a strong capacity to enhance vascular density, promote stable neovascularization, and improve long-term cardiac function. As the field moves forward, optimizing these approaches for human application will require continued refinement of delivery techniques, dosing strategies, and long-term safety assessments.

## LIMITATIONS AND FUTURE DIRECTIONS FOR mRNA THERAPEUTICS

Although mRNA therapies hold immense potential, several limitations must be addressed to enable widespread clinical application. One major challenge is the lack of precise temporal control over protein expression due to the inherently transient nature of mRNA. Although transient expression proves beneficial for minimizing long-term risks associated with certain genes, it can also limit sustained therapeutic effects, requiring repeated dosing which may not always be feasible or cost-effective. Another key hurdle is the lack of absolute specificity in targeting, even with advanced cell-type-specific translation strategies such as SMRTs. As mRNA expression is not entirely restricted to the intended cell population, some degrees of leakiness and off-target effects remain. This underscores the need for more precise delivery mechanisms that enhance tissue and cell-type specificity.

Immunogenicity and delivery-associated toxicity also remain significant concerns. While incorporating modified nucleotides like N1-methylpseudouridine can weaken immune detection, LNPs can intrinsically trigger pro-inflammatory responses. LNPs are recognized by pattern recognition receptors (PRRs), triggering the release of pro-inflammatory cytokines such as 1L-1β, IL-6, and type I interferons. This response raises safety concerns in therapeutic applications, where sustained inflammation could worsen cardiac injury. Furthermore, LNP components such as PEG lipids have been implicated in hypersensitivity reactions, including IgE-mediated anaphylaxis and complement activation-related pseudo allergy (CARPA)^[80]^. Addressing these risks will require next-generation delivery vehicles that promote efficient endosomal escape while minimizing innate immune activation, as well as careful optimization of mRNA chemistry, lipid composition, and dosing regimens.

Future research should focus on refining mRNA delivery systems to improve endosomal escape, enhance biocompatibility, and reduce off-target expression and immune activation. Additionally, cost-effective and scalable manufacturing techniques are needed to facilitate clinical-grade mRNA production, particularly with the use of N1-methylpseudouridine. Another clinical research priority is addressing long-term safety concerns, including the potential for unintended immune responses or cumulative toxicity with repeated dosing. More robust preclinical and clinical studies are required to assess the durability of therapeutic effects and the impact of chronic mRNA administration in human patients. Advances in personalized medicine, such as patient-specific mRNA constructs tailored to individual genetic and immune profiles, hold promise for increasing efficacy while minimizing adverse effects.

In addition to addressing these technological and logistical hurdles, future research should continue investigating and developing the targets discussed in this review as well as the discovery of other targets and pathways relevant to cardiac protection, regeneration, and vascular regeneration. Exploring innovative combinations of therapeutic factors, adaptive dosing strategies, and synergistic multi-gene approaches could unlock greater regenerative potential. Furthermore, a major unmet need remains the development of systemic delivery systems capable of selectively targeting the heart, as current approaches still rely heavily on direct intracardiac injection, limiting both practicality and scalability for human applications.

By addressing these challenges, mRNA therapeutics have the potential to revolutionize the treatment landscape for IHD and beyond, offering a powerful platform for precision medicine and regenerative therapies.

## CONCLUSIONS

mRNA-based therapies hold significant potential for advancing treatments in IHD by addressing key limitations in viral vector-based and recombinant gene techniques. By categorizing mRNA methods according to their focus on cardiac regeneration, cardiac protection, or cardiovascular regeneration, this review provides a structured resource for exploring the therapeutic applications of mRNA in cardiac medicine. ModRNA is particularly advantageous due to its reduced immunogenicity, which allows for safer and more effective protein expression in targeted tissues. Innovative approaches such as the SMRTs system for cell type-specific delivery systems as well as active and passive LNP targeting enhance the precision and safety of these therapies. As a valuable tool in the therapeutic manipulation of cellular processes, transient mRNA-based protein expression enables temporal and spatial control over pathways involved in tissue repair and regenerative responses without the risks associated with sustained expression. Altogether, these advancements underscore mRNA therapies as a promising and precise way to tackle the complex challenges of IHD.

## FUNDING

This work was funded by NIH grants R01 HL142768-01 and R01 HL149137-01.

## AUTHOR CONTRIBUTIONS

MA provided a comprehensive overview of the preclinical and clinical studies mentioned in this review, created the figure, and composed the manuscript. IT contributed to the formatting of publications reviewed into tabular form for Table 1. LZ reviewed and approved the final manuscript.

## CONFLICT OF INTEREST STATEMENT

This literature review did not involve human or animal subjects and therefore did not require ethical approval. The authors declare that they have no conflict of interest with regard to the content of this manuscript.

## DATA SHARING STATEMENT

All data generated or analyzed during this study are included in this published article
